# Missed Giant Lower Esophageal Leiomyoma in a Young Female Presenting with Refractory Gastroesophageal Reflux Disease

**DOI:** 10.1155/2021/9925224

**Published:** 2021-07-20

**Authors:** Duc Trong Quach, Luu Huy Le, Quy-Dung Dang Ho

**Affiliations:** ^1^Department of Internal Medicine, University of Medicine and Pharmacy at Ho Chi Minh City, Ho Chi Minh, Vietnam; ^2^Department of Gastroenterology, Nhan Dan Gia Dinh Hospital, Hochiminh, Vietnam; ^3^Department of General Surgery, University of Medicine and Pharmacy at Ho Chi Minh City, Ho Chi Minh, Vietnam; ^4^Department of Endoscopy, Cho-Ray Hospital, Hochiminh, Vietnam

## Abstract

Esophageal leiomyoma is a rare disease commonly reported in middle-aged patients with a male predominance. Many patients are asymptomatic, and a few may present with symptoms such as dysphagia and chest pain. However, heartburn is only reported in patients with accompanying hiatal hernia. We hereby report a giant lower esophageal leiomyoma with concomitant hiatal hernia in a young Vietnamese female, who presented with refractory gastroesophageal reflux symptoms. The diagnosis was challenging as the tumor grew outward. As a consequence, the patient did not experience dysphagia and the tumor was hardly detected under endoscopy. The hiatal hernia in this patient was probably related to the presence of the leiomyoma. It is important to look carefully for submucosal tumor at the lower esophagus and cardia under endoscopy in patients with similar manifestations.

## 1. Introduction

Esophageal leiomyoma is a rare disease, which is commonly reported in patients aged 40 to 50 years with a male-to-female ratio of 2 : 1 [1–3]. About a half of patients with esophageal leiomyoma are asymptomatic [1]. Also, the majority of patients experience symptoms only if the tumor is large. The mean tumor diameter among symptomatic patients is about 5.3 cm, as compared to 1.5 cm in asymptomatic patients [3]. The most common symptom is dysphagia followed by chest pain while heartburn is only reported in patients with accompanying hiatal hernia [1, 2]. We hereby report a giant lower esophageal leiomyoma with concomitant hiatal hernia in a young female patient, who presented with refractory gastroesophageal reflux disease (GERD). The diagnosis was challenging as the tumor protruded only a little into the esophageal lumen. As a consequence, the patient did not experience dysphagia and the tumor was hardly detected under endoscopy.

## 2. Case Report

**Figure 1:**
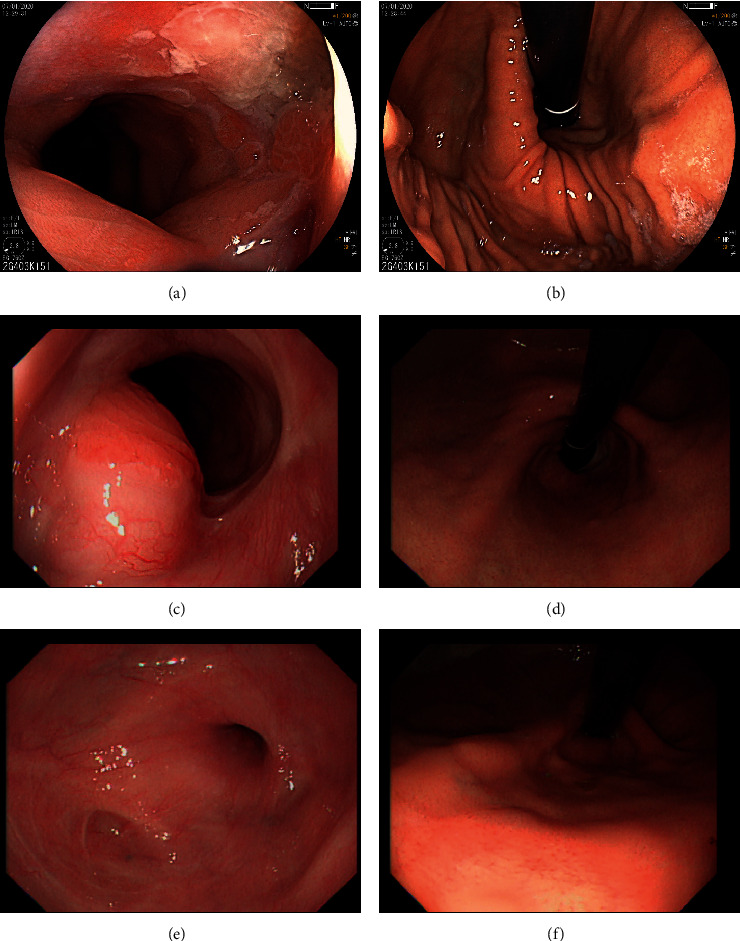
The esophageal ulcer just above the gastroesophageal junction and hiatal hernia detected during the first upper astrointestinal endoscopy (UGIE) (a, b). The ulcer had completely healed after 10-week treatment with double-dose proton pump inhibitor. A protrusion at the cardia, which was only identified on the look down view with minimum insufflated air, suspecting submucosal lesion is shown (c, d). The UGIE performed four months after her surgery demonstrated good results of Nissen fundoplication (e, f).

A 28-year-old female patient suffered from postprandial lower chest pain and heartburn for more than one year. Her past medical history and family history were unremarkable. She lost 4 kilograms and did not experience dysphagia or regurgitation. Three months before her visit to our hospital, the pain had become more severe. The upper gastrointestinal endoscopy (UGIE) performed at another hospital showed a lower esophageal ulcer, which was located just above the gastroesophageal junction, hiatal hernia, and antral-predominant gastritis (Figures 1(a) and 1(b)). The *H. pylori* antibody test was positive, and the histological result of esophageal ulcer was benign. The patient received *H. pylori* eradication therapy followed by a regimen of proton pump inhibitor (PPI) at a standard dose in combination with antacid for more than 8 weeks. However, her pain did not resolve and she sought for medical consultation in our hospital.

We managed the patient as a case of refractory GERD. At first, the patient was treated with rabeprazole 20 mg bid in combination with alginate-antacid at bedtime for 2 weeks. The pain improved but was still intolerable and badly affected her sleep quality. The regimen was switched to rabeprazole 20 mg bid in combination with amitriptyline 12.5 mg at bedtime, which resulted in a complete clinical response and was continued for 10 weeks. The dose of rabeprazole was then tapered to 20 mg qd aiming to stop completely before UGIE was performed to document the efficacy of treatment. However, the pain recurred badly after about a week. The patient requested us return to double-dose rabeprazole, which again relieved the pain.

**Figure 2:**
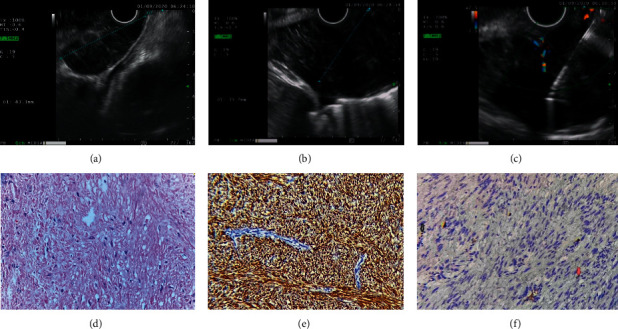
Endoscopic ultrasound showed that the submucosal tumor arose from the fourth layer of the esophageal wall (a, b). EUS-FNA was performed (c). The histological findings on hematoxylin and eosin staining (d) and immunohistochemical stainings with Desmin (+) and Ki67 (+) confirmed that the tumor was a leiomyoma.

**Figure 3:**
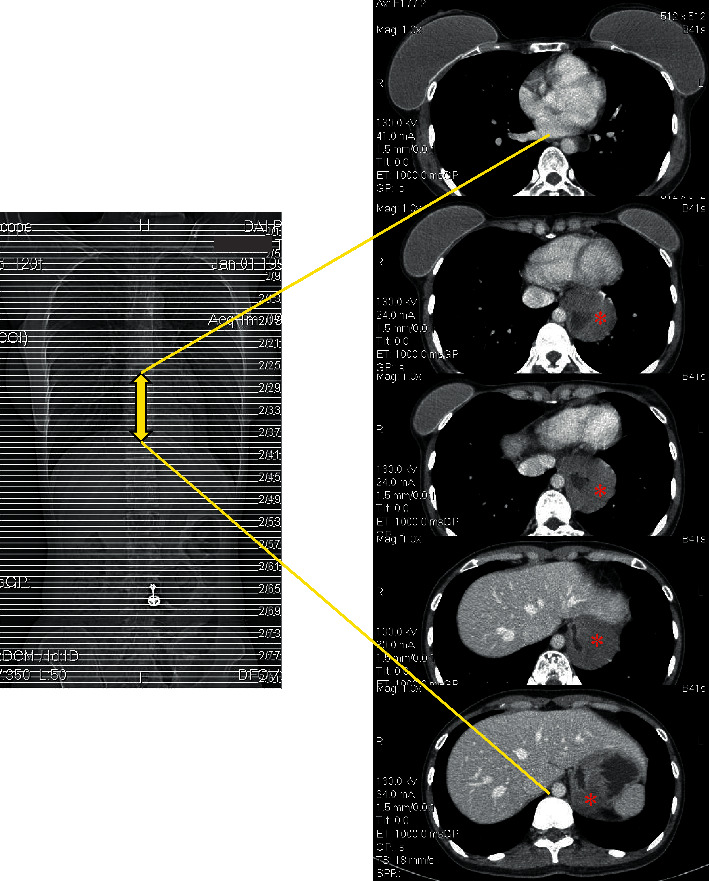
Uneven thickening of the distal esophagus and cardia on the CT scan, measuring about 8 cm in length and 3.5 cm in diameter. The asterisks mark the tumor mass.

The second UGIE performed when the patient was still on PPI showed that the lower esophageal ulcer had completely healed (Figures 1(c) and 1(d)). There was a protrusion at the cardia, which was only identified when air was minimum insufflated, suspected of a submucosal lesion. Endoscopic ultrasound (EUS) and CT scan were performed which surprisingly showed a giant submucosal tumor at the fourth layer of the lower esophagus and the cardia (Figures 2 and 3). EUS-FNA was performed, and the histological examination confirmed that this lesion was a leiomyoma (Figure 2).

**Figure 4:**
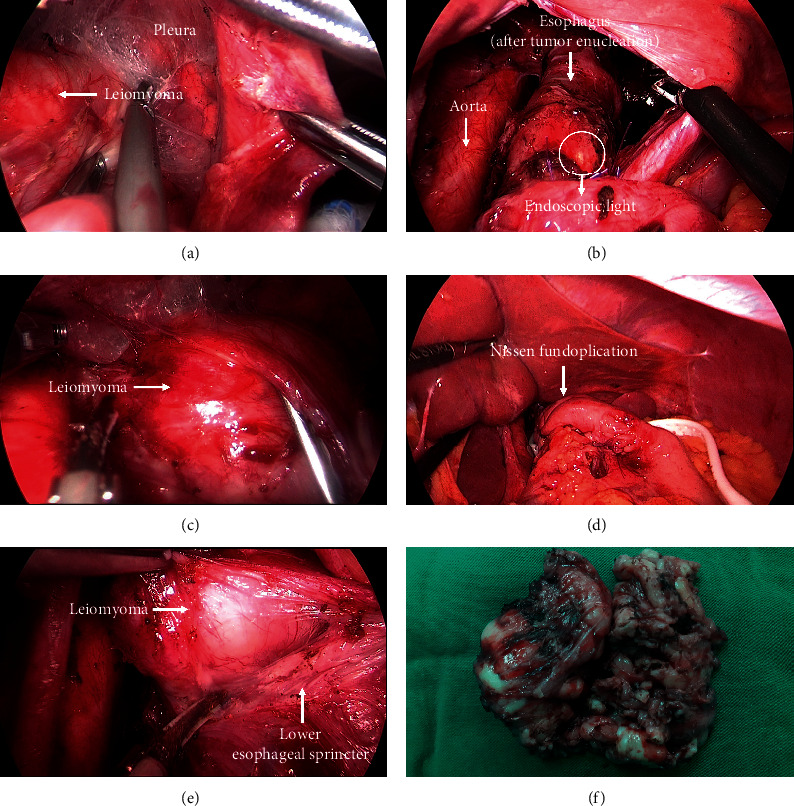
The distal esophagus evolving leiomyoma was mobilized from the mediastinal pleura and structures (a, b). The leiomyoma was carefully dissected out of the esophagus (c). The tumor had been completely removed (d). Nissen fundoplication was performed (e). The postoperation specimen is shown (f).

The patient was performed laparoscopic transhiatal surgery to enucleate the esophageal leiomyoma. In addition, hiatal hernia was repaired with laparoscopic Nissen fundoplication (Figure 4). As the tumor is too large, it was cut into pieces to facilitate its removal. The solid straightened tumor was approximately 8 cm × 3 cm in size (Figure 4). The patient made an uneventful postoperative recovery. During the first two weeks, she had experienced dysphagia and regurgitation, which have resolved completely.

Four months after her surgery, UGIE was performed again which showed good results of fundoplication surgery (Figures 1(e) and 1(f)). The patient sometimes had epigastric pain which could be completely controlled with intermittent rabeprazole therapy at a standard dose.

## 3. Discussion

The clinical presentation of this patient would suggest dyspepsia be a differential diagnosis as postprandial pain is the predominant symptom. However, the first UGIE clearly showed an esophageal ulcer accompanying hiatal hernia. This suggested that the pain be an atypical symptom of GERD. According to the Asia-Pacific consensus, reflux symptoms which respond poorly to PPI at a standard dose in 8 weeks are considered as refractory GERD [4]. This patient satisfied this definition, and she was managed as refractory GERD. The lower esophageal ulcer as a complication of GERD and the presence of hiatal hernia were the two factors suggesting that acid suppression not be enough in this patient.

The findings on EUS and CT scan and intraoperative observation all showed that the tumor did not penetrate the mucosal layer. The esophageal ulcer could be due to tumor compression, which caused anatomical changes weakening the antireflux barrier. That the giant tumor was missed during the first UGIE would be due to some reasons. First, this leiomyoma tumor did not protrude much into the esophageal lumen. Therefore, dysphagia was absent and its endoscopic detection required a high suspicion with minimum air insufflation. There have been some case reports showing that esophageal leiomyoma might even masquerade as achalasia [5, 6]. Dysphagia was the predominant symptom in these patients, and UGIE revealed a dilated and tortuous esophagus with no clear findings suggesting of submucosal tumor. Second, the patient is a young female while leiomyoma has been generally reported in middle-aged, male patients [1–3]. A few case reports in Asia previously described giant esophageal leiomyoma in young females, but the diagnosis of these cases were not challenging as dysphagia was a predominant symptom and the tumors were easily identified under endoscopy [7, 8]. This is the first time that such a giant leiomyoma is reported in a young woman in Vietnam.

There were several options for the enucleation of such tumors, such as the thoracotomic, thoracoscopic, or laparoscopic approach [9, 10]. However, the laparoscopic transhiatal approach was chosen in order to avoid causing extensive defect in the esophageal wall, which might lead to impaired esophageal peristalsis and damage of the lower esophageal sphincter. Also, the hiatal hernia was finally repaired with laparoscopic Nissen fundoplication after finishing the enucleation procedure. Recently, submucosal tunneling endoscopic resection has been introduced into the management of subepithelial lesions of the esophagus. Using meticulous dissection techniques, en bloc resection of giant esophageal leiomyoma could be performed safely and effectively in the hands of experts [11].

This case raises some interesting questions. First, whether the pain in this patient was due to tumor compression or cardia ulceration or it was actually an atypical reflux symptom. As the frequency and severity of the pain paralleled with the intensity of acid suppression therapy, acid reflux would be the major pathogenesis factor. But, that the pain was completely controlled with a neuromodulator add-on therapy suggested that tumor compression be a contributory factor. The weak point of this case was that there was no esophageal manometry and pH monitoring investigations to document the symptom index. However, that the pain significantly improved after tumor removal and hiatal hernia repair and only intermittent standard-dose PPI therapy was enough to control the patient's symptoms supported this theory. Second, whether there was a direct association between the presence of leiomyoma and hiatal hernia or this coexistence was by chance in this patient. There have been several studies showing that the coexistence between hiatal hernia and lower esophageal submucosal tumor was very common [1, 7, 12, 13]. A case series on 66 patients who underwent surgery therapy for esophageal leiomyoma found that hiatal hernia presented in 15 (23%) patients [13]. The association may be incidental, but there is also suggestion that the tumor pulls the esophageal-gastric junction and causes hiatal hernia [7]. In Vietnam, the prevalence of hiatal hernia was only 2.3% among patients who presented with upper gastrointestinal symptoms [14]. Also, it was especially very uncommon in such a young female. Therefore, we believe that the hiatal hernia could be associated with the leiomyoma in this patient.

## 4. Conclusions

We report a case of giant lower esophageal leiomyoma with concomitant hiatal hernia in a young female, who presented with refractory gastroesophageal reflux symptoms. The leiomyoma was missed during the first UGIE as the tumor grew outward. It is important to look carefully for submucosal tumor at the lower esophagus and cardia under endoscopy in patients with similar manifestations.

## Data Availability

No data were used to support this study.
